# Development and child health in a world of synthetic chemicals

**DOI:** 10.1038/s41390-024-03547-z

**Published:** 2024-09-14

**Authors:** Jessica L. Wager, Jennifer A. Thompson

**Affiliations:** 1 Cumming School of Medicine, University of Calgary, Calgary, Alberta Canada; 2 Libin Cardiovascular Institute, Calgary, Alberta Canada; 3 Alberta Children’s Hospital Research Institute, Calgary, Alberta Canada

## Abstract

**Abstract:**

Chemical pollution is one of today’s most significant threats to the developmental potential of children worldwide. Maternal exposure to toxicants can perturb sensitive windows of fetal development, indirectly through promoting antenatal disorders, abnormal placental adaptation, or directly through maternal-fetal transport. Current evidence clearly shows that persistent organic chemicals promote hypertensive disorders of pregnancy, placental abnormalities, and fetal growth restriction, whereas findings are less consistent for phthalates and bisphenols. Prospective birth cohorts strongly support a link between adverse neurodevelopmental outcomes and prenatal exposure to flame retardants and organophosphate pesticides. Emerging evidence reveals a potential association between in utero exposure to bisphenols and childhood behavioral disorders, while childhood metabolic health is more consistently associated with postnatal exposure to phthalates and bisphenols.

**Impact:**

- Synthesizes emerging evidence linking modern forms of chemical pollution to antenatal disorders, fetal growth restriction and childhood disorders.
- Highlights potential developmental impacts of emerging pollutants of concern now ubiquitous in our environment but without regulatory restrictions.

## Statement of problem

Calls for urgent action to address the ecological crisis are growing amid mounting threats to ecosystems, political stability and human health. In 2022, the United Nations General Assembly signed a landmark resolution that recognizes access to a clean, healthy and sustainable environment as a universal human right, which may catalyze action tackling climate change, loss of biodiversity, and chemical pollution.^1^ The *Lancet* Commission on Pollution and Health estimated that 9 million premature deaths worldwide were attributable to pollution, drawing attention to the significant impact of pollution on global health and healthcare costs.^2^ The human health toll of pollution is largely driven by intensification of current epidemics of non-communicable diseases (NCDs), which are top causes of disability and death worldwide. In the most highly polluted countries, pollution-related deaths from cardiovascular disease, cancer and other NCDs are on par with deaths caused by smoking.^3^ While significant progress has been made in controlling certain forms of pollution with established links to disease, overall deaths due to pollution have increased over the past several decades. The annual global production of synthetic chemicals is ~2.3 billion tonnes and is expected to double by the year 2030.^4^ According to the Environmental Protection Agency (EPA), only 1% of chemicals present in household products have undergone rigorous testing for safety, and each year 10 million new chemicals are manufactured and released into the environment.^5^ Since emerging toxicants are not taken into account in estimates of pollution-related deaths, the true cost of pollution to human health and well-being is likely underestimated.

## Modern pollutants of concern

A challenge to addressing environmental determinants of health noted in the *Lancet* Commission is the release of a large number of new synthetic chemicals with unknown health effects, which creates a lag in time between widespread dispersion and the recognition of harmful effects. In many cases, identification and regulation of hazardous chemicals have been followed by replacement with “regrettable substitutions” that are themselves harmful, resulting in a failure of regulatory action to protect public health. A historical example is the replacement of highly toxic arsenic-based pesticides with the organochloride pesticide dichlorodiphenyltrichloroethane (DDT), which was celebrated as a ground-breaking solution to pest control until the publishing of Rachel Carson’s *Silent Spring* in 1962 documenting the accumulation of DDT in the environment and food chain and its deleterious impact on wildlife and human health. A more recent example that garnered public attention is the replacement of bisphenol A (BPA) with synthetic analogs such as bisphenol F (BPF), bisphenol AF (BPAF), and bisphenol S (BPS) in the production of polycarbonate plastics and epoxy resins. Production and exposure to these substitutes have increased since Canada, the United States, and European countries banned the use and import of BPA in baby products.^6^ Emerging data suggest BPA substitutes to exhibit the same endocrine disrupting properties and health impacts as their predecessor, yet these widely dispersed chemicals are not covered under recent proposals by the European Safety Authority to reduce the tolerable daily intake (TDI) of BPA.^7^ Following a similar storyline, PFOA, a persistent chemical best known for its use in non-stick cookware, was replaced with other chemicals belonging to the group of per and polyfluoroalkyl substances (PFAS) that did not live up to their promise of more environmentally friendly and healthy alternatives.

While the case of BPA and PFOA are notorious examples brought to light by the popular media, there are many other examples of replacement chemicals for which evidence for environmental and human harm is accumulating or for which data on human health is lacking. Polybrominated diphenyl ethers (PBDEs) are persistent organic chemicals belonging to the class of flame retardants that became widespread in the environment due to their use in the manufacture of furniture, infant products and electrical devices after the banning of polychlorinated biphenyls (PCBs) and polybrominated biphenyls (PBBs) in the 1970s. Over the past two decades, PBDEs have been phased out in most countries on account of their accumulation in the environment and toxic properties and are now substituted with organophosphate ester flame retardants (OPFRs). While OPFRs are less persistent in the environment with a short half-life, their chemical volatility and solubility make them vulnerable to leaching out into the environment and they are now universally detected in human samples.^8^ Phthalates are a family of synthetic organic chemicals used in plastics, fragrances, and cosmetics. Although phthalates are rapidly metabolized, they are ubiquitous in the environment and human exposure is universal, with the major route of exposure being contamination of food for high molecular weight phthalates and personal care products for low molecular weight phthalates.^9^ In recent years, many countries have limited the use of several phthalates (e.g. DEHP, DBBP, BBP), with most regulations targeting child products. Canada and some European countries have added to the list of phthalates that are regulated, while several new phthalate substitutes have been released to the market. The above illustrate that the capacity and reactive approach of regulatory mechanisms fail to safeguard the public from health harms of an increasingly toxic environment.

The impact of chemical pollution on human health is most palpable in cases of occupational exposure or in “toxic hotspots,” communities located in close proximity to manufacturing or waste disposal facilities. It is more difficult to measure the insidious health toll of ubiquitous exposure of the majority of the population to the wide range of synthetic chemicals found in numerous household products and food and beverage containers. Health impacts at both the individual level and population level are often the most severe when exposure occurs during sensitive windows of development in early life. In this review, we synthesize current evidence for emerging threats that modern chemical pollutants pose to the developing fetus and child.

## Sensitive windows of development in early life

Chemical pollution poses the greatest threat to human health when exposure occurs during early life. Since the developmental origins of health and disease (DOHaD) hypothesis was conceived in the early 1990s, there has amassed a large body of data highlighting a critical role for the early life environment in shaping lifelong health and risk for NCD. Early life sets the trajectory of lifelong health because organ structure and function are to a large extent determined during windows of development in prenatal life and early postnatal life. Doses of toxic chemicals that are harmless in adulthood can have a discernible impact when coinciding with the rapid cellular proliferation, differentiation and tissue accretion that occurs in windows of organ development. A subtle effect on fetal and child development at the individual level magnifies at the societal level when a significant proportion of the population is exposed. This is illustrated by the case of lead, a neurotoxic heavy metal that became pervasive in the environment in the early twentieth century and is associated with poor neurodevelopmental outcomes in children at low levels of exposure.^10^ After passing of the Lead-based Paint Prevention Act in the 1970s, followed by the Clean Air Act which banned leaded gasoline in the 1990s, blood levels of lead in school-aged children declined by over 80% and average IQ scores in children born in the 90s were on average 2.2–4.7 points higher than children born in the preceding decades.^11^ Regulatory action to reduce exposure to lead is one of history’s most notable public health accomplishments. Today, modern forms of chemical pollution that are widespread in the environment exhibit neurotoxicity, reproductive toxicity, and endocrine disrupting properties that have the potential for persistent negative effects when disrupting key developmental events.

**Fig. 1:**
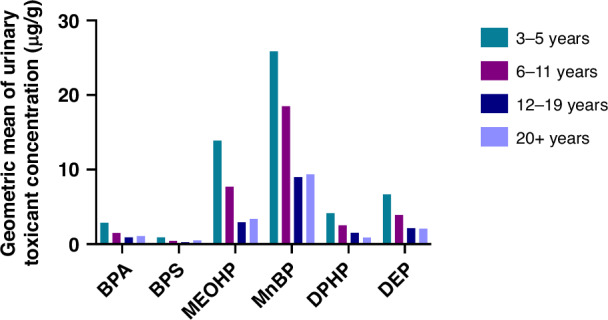
Exposure levels of common chemical pollutants in children and adults in the USA. Geometric mean of urinary concentrations (in µg/g of creatinine) of common chemical pollutants for children ages 3–5, 6–11, 12–19, and adults 20+ years of age in the U.S. population, derived from the 2015–2016 National Health and Nutrition Examination Survey (NHANES). Toxicants included: bisphenol A (BPA), bisphenol S (BPS), MeOHP (Mono(2-ethyl-5-oxohexyl) phthalate), a metabolite of mono(2-ethylhexyl) phthalate, MnBP (Mono-n-butyl phthalate), DPHP (flame retardant metabolite), and DEP (metabolite of several organophosphorus insecticides).

Susceptibility to environmental exposures during the early life window of development is heightened by immature organs and detoxification defences prior to birth and the physiological characteristics and behaviors unique to infants and children. In the first two months of life, the metabolism and renal clearance of toxicants is lower compared to adulthood, leading to higher bioaccumulation of toxicants.^12^ Relative to body weight, respiration rate, food intake and skin surface area are higher in infants and children compared to adults, increasing the dose of inhaled, ingested, or absorbed toxicants per unit body mass.^12,13^ Children and infants display unique behaviors such as oral sensory seeking that expose them to higher levels of certain pollutants compared to the adult population. For instance, young children have higher exposure to BPA and other ubiquitous chemicals compared to older age groups according to National Health and Nutrition Examination Survey (NHANES) biomonitoring data (Fig. 1). Therefore, in early life, susceptible periods of development coincide with periods along the life course wherein the risk of exposure to certain chemicals is higher and detoxification defences are immature.

## Adaptations to pregnancy

Exposure to chemical pollutants may compromise the ability to maintain a healthy pregnancy and increase the risk for pregnancy complications. During pregnancy every major organ system in the body undergoes adaptations to support conception and fetal development and prepare for parturition and lactation. These maternal adaptations to pregnancy are orchestrated by hormones, produced mainly by the ovaries and placenta. Thus, endocrine disrupting chemicals (EDCs) have the potential to interfere with hormone-driven maternal adaptations to pregnancy and compromise the ability of the female reproductive system to initiate and maintain a healthy pregnancy to support optimal fetal growth and development. Antenatal complications are associated with adverse perinatal outcomes like abnormal fetal growth or preterm birth and can compromise lifelong health and disease risk in the offspring. There is some evidence for a link between exposure to EDCs and hypertensive disorders of pregnancy including preeclampsia^14^; however, findings are inconsistent. A review of the evidence by Khan and Trasande concluded that the link between EDCs and hypertensive disorders of pregnancy are strongest for heavy metals, including lead and cadmium, as well as organochlorine pesticides and PCBs, which have been regulated or phased out.^15^ As far as emerging toxicants, positive associations between preeclampsia and urinary levels of BPA or phthalates measured during gestation have been reported.^15^ The Health Outcomes and Measures of Environment (HOME) study prospectively collected urine at 16 and 26 weeks of gestation and found that hypertension during pregnancy was more likely to occur in women with the highest prenatal levels of mono-benzyl phthalate.^16^ In a prospective birth cohort, low circulating levels of placental growth factor-1 (PlGF) and high levels of soluble fms-like tyrosine kinase 1 (sFlt-1), a biomarker of preeclampsia, were associated with higher urinary levels of BPA and metabolites of the phthalate di-ethylhexyl (DEHP) throughout pregnancy.^17^ In another prospective birth cohort, the R Generation Study in the Netherlands, higher urinary concentrations of high molecular weight phthalate metabolites in early pregnancy were associated with increased ratios of sFlt-1/PlGF, while high concentrations of total bisphenols including new BPA substitutes predicted higher pulsatility index of the umbilical artery at mid-gestation.^18^ However, neither phthalate metabolites nor bisphenols were associated with hypertension during pregnancy.^18^ A recently published meta-analysis reported that although some studies show preeclampsia and gestational hypertension to be correlated with phthalates and bisphenols, overall, the evidence is weak and inconclusive.^19^ There is strong evidence for a link between gestational diabetes and exposure to persistent pollutants like PCBs and PBDEs; however, emerging toxicants such as those derived from plastics and personal care products have been studied less in this context.

## Placental transport and function

The placenta is essential to maintenance of pregnancy and development of the fetus, secreting hormones to regulate maternal adaptations, mediating maternal-fetal transport of oxygen and nutrients, and acting as a barrier to protect the fetus from potentially harmful agents. Human biomonitoring studies have detected a number of synthetic chemicals in the serum or urine of pregnant women, as well as in breast milk, umbilical cord blood, placentae, amniotic fluid and meconium.^20^ In pregnant women enrolled in the HOME study, a median of 44 chemicals were detectable in all 175 urine or serum samples, among the measured phthalate metabolites, BPA, PCBs, PBDEs, organochlorine pesticides and PFAS.^21^ In healthy mid-gestation pregnant women, 5 of 12 PFAS measured were detected in over 80% of serum samples with the legacy compounds PFNA, PFOA and PFOS found at the highest concentrations and detected in 100% of samples.^22^ The same study found that 83-99% of samples had detectable levels of OPFRs, substitutes used to replace PBDEs. Detection of these chemicals in the placenta, cord blood, and amniotic fluid, suggest transfer across the placenta, while detection in meconium may be due to swallowing of contaminated amniotic fluid that occurs in the third month of pregnancy. The placenta serves as a barrier between mother and fetus that expresses enzymes and membrane transporters to protect the fetus from xenobiotics. However, the placental barrier is not impenetrable, and toxicants can reach the fetal compartment to various degrees depending on metabolism by the placenta and whether the route of transplacental transfer is via passive diffusion or protein transporters. The best method to study placental transport of xenobiotics is the ex vivo perfused human placenta. A recent study using this model assessed 15 bisphenols and showed some to cross the placenta by passive diffusion, while the transport of others was limited.^23^ Compared to the maternal circulation, the half-life of bisphenols is 20-fold higher in the fetal compartment due to immature detoxification defences and slower clearance.^24^ Grandin et al. measured bidirectional transport in the human placenta and demonstrated that the placental barrier was less efficient at protecting the fetus against exposure to BPA compared to BPS.^24^ Despite slower placental transport, BPS accumulated to a greater extent in the fetal compartment compared to BPA, as shown in a pregnant sheep model.^25^ Persistent pollutants such as PFASs and PBDEs accumulate in the placenta and have been measured in the umbilical cord blood and in breast milk, suggesting early life exposure and increased bioaccumulation potential.^26,27^ Ruis et al. showed that in both rats and humans, PBDEs accumulate to a greater extent in the fetal compartment of the placenta, suggesting active placental transport.^28^ OPFRs have been shown to accumulate in the placenta and are detected at levels equaling or exceeding that of PBDEs.^29^

Maternal exposure to toxicants can impact fetal development indirectly through impairing placental development and function. Indices of aberrant placental development such as a reduction in placental size, reduction in the junctional zone and impairment of spiral artery remodeling have been measured in pregnant rodents exposed to bisphenols or phthalates.^30^ A recently published study showed that the new generation bisphenol, BPS, had a similar impact on placental structure and gene expression compared to its predecessor, BPA. In another recent study, placenta lesions in pregnant CD1 mice were found to occur due to exposure to either PFOA or the PFOA replacement known as GenX.^31^ In human mid-gestation pregnancies, markers of placental stress and abnormal cytotrophoblast invasion were positively associated with levels of PFAS and to a lesser extent, OPFRs.^22^ Interference in normal placental development and function may be an important mechanism by which exposure to chemical pollutants during pregnancy increase the risk for antenatal complications such as preeclampsia and adverse outcomes like impaired fetal growth or preterm birth.

## Neonatal outcomes

Aberrant fetal growth leading to small or large-for-gestational age infants reflects a suboptimal intrauterine environment and is associated with heightened risk for a number of childhood diseases as well as chronic diseases later in life including type 2 diabetes and cardiovascular disease. Evidence in human cohorts suggest an impact of EDCs and other chemical pollutants on fetal growth, although findings are inconsistent. The persistent organic pollutants are most strongly and consistently associated with abnormalities in fetal growth. A recent nested case control study collected serum at 10-28 weeks of gestation and found that exposure to a mixture of PFAS, PCBs, and organochlorine pesticides were associated with a small decrease in birth weight and crown-to-heel length, with PFAS most strongly contributing to the relationship.^32^ A systemic review of human epidemiological studies concluded that there was sufficient evidence supporting a link between prenatal PFOA exposure and indices of reduced fetal growth, estimating a decrease of 19 grams in birth weight for each 1 ng/mL increase in PFOA exposure.^33^ A more recently published meta-analysis by Gao et al. reported that intrauterine growth restriction was more common in pregnant women in the category of highest PFOA and PFOS exposure but did not find an overall association with small-for-gestational age or large-for-gestational age.^34^ There was a positive linear association between PFOS and preterm birth. Koustas et al. undertook a meta-analysis of mouse studies that used oral gavage to expose pregnant dams to PFOA during pregnancy and found a significant reduction in pup birth weight.^35^ In a cohort of 604 healthy pregnant women, EDC mixtures dominated by PBDEs, and organochlorine pesticides were predictive of small birth weight, while higher levels of PCBs were associated with higher birth weight.^36^ The ECHO multi-cohort study that included 3339 pregnant women, demonstrated a negative relationship between exposure to PFAS and birth weight.^37^ Very little research has investigated birth outcomes associated with chemicals used to replace PFAS; however, a study performed in pregnant rats showed that exposure to GenX led to a reduction in birth weight of the pups.^38^ Maternal samples of BPA, BPS, and BPF, were taken in the first, second and third trimesters in 1379 women in the Generation R Study. While high maternal BPS levels were related to a larger fetal head circumference and weight, there was no relationship between total bisphenols and indices of fetal growth or preterm birth.^39^ A prospective birth cohort study measured urinary bisphenols and phthalate metabolites from mid-gestation to late-gestation in 855 mother-fetal pairs and found phthalate exposure decreased or increase fetal size depending on the sex of the fetus.^40^ Meta-analyses have concluded that there is insufficient evidence to support a link between bisphenol exposure and fetal growth trajectories.^41^ Yang et al. quantified pesticide levels in maternal blood where organochlorides were detected in over 80% of samples. Increased levels of the organochlorine pesticide β-hexachlorocyclohexane (β-HCH) were found associated with decreased birth weight in 1039 mother-infant pairs.^42^ A longitudinal study reported that a one-unit increase in exposure to an EDC mixture containing PFAS, PCBs, organochlorine pesticides, phthalates, and phenols was associated with a decrease in birth weight and slower early postnatal growth.^43^

## Neurodevelopment

Development of the nervous system starts with formation of the neural tube in the 3^rd^ and 4^th^ week of gestation, followed by neurogenesis, differentiation, synaptogenesis, and myelination in embryonic and fetal life.^44^ Brain development continues into the postnatal period with critical periods of synaptogenesis, synaptic pruning, and myelination in childhood. Plasticity during early life brain development confers a high capacity for recovery from environmental insults; yet even mild perturbations in fetal growth trajectories or low exposure to environmental toxicants can program observable deficits in cognitive function and increase the risk for behavioral and psychiatric disorders in later life. Exposure to chemical pollutants leading to neurodevelopmental deficits, behavioral abnormalities and challenges in executive function may arise indirectly from perturbed fetal growth or directly by penetrating the blood brain barrier, which is functional as early as 8 weeks gestation.^45^

Developmental neurotoxicity of lead, arsenic, DTT, PCBs and other historical environmental pollutants has been well established by decades of research. Emerging evidence suggests that modern forms of pollution like EDCs interfere with neuronal proliferation, migration, synaptogenesis and myelination in the developing fetal brain, potentially through hormonal dysregulation, disruption of neurotransmitter uptake and release, or direct toxicity.^44,46^ A disruption in the synthesis, transport and metabolism of thyroid hormones have been shown to occur with exposure to PBDEs and organophosphate esters released into the environment by OPFRs, as well as BPA, phthalates and their new generation substitutes.^44,46,47^ Evidence also suggests disruption in calcium, dopamine, or other neurotransmitter homeostasis may underlie the mechanism by which EDCs lead to behavioral disorders.^48,49^

There exists strong epidemiological evidence linking early life exposure to flame retardants with impaired cognition and behavior in childhood, with sex-dependent behavioral differences in some cases. Two studies examined > 300 mother-child dyads from the HOME Study and found increased hyperactivity, and a tendency towards externalizing problems in children born to mothers with high PBDE exposure.^50,51^ Further, these studies reported a 4.1–4.5-point deficit in Full Scale IQ (FSIQ) for every 10-fold increase in prenatal exposure to PBDE. Another prospective birth cohort ( > 300 children) demonstrated an inverse association between exposure to PBDEs *in utero* or childhood and cognitive abilities as assessed by FSIQ and verbal comprehension.^52^ Studies investigating currently used flame retardants, OPFRs, are very limited. However, data from the Pregnancy, Infection and Nutrition (PIN) Study demonstrated that urinary levels of certain OPFR metabolites during pregnancy were related to cognitive and behavioral deficits in early childhood.^53,54^

In several prospective birth cohort studies, increased risk for behavioral problems and cognitive impairments have been reported in children born to mothers with higher exposure levels of bisphenols during pregnancy. For instance, Braun et al. found a 5.9-point increase in externalizing scores for every 10-fold increase in maternal urinary BPA during pregnancy in girls, but not boys, and this association remained consistent between 2 and 8 years of age.^50^ Prenatal exposure was shown to have no impact on language abilities and no correlations were reported between postnatal BPA exposure and child behavioral score.^50^ In a small cohort (98 mother-child pairs), magnetic resonance imaging (MRI) revealed less developed white matter in school-aged children born to mothers who had higher levels of urinary BPA measured during the second trimester of pregnancy and may show alterations in white matter microstructure.^55^ Overall, human observational studies suggest the prenatal period to be a window of susceptibility to the neurodevelopmental impact of BPA. Although far fewer studies have investigated new BPA-replacements, emerging evidence reveals potential neurodevelopmental effects. Recently, a study using the Swedish Environmental Longitudinal Mother and Child, asthma and allergy cohort measured maternal urinary levels of BPA, BPF and BPS during pregnancy and found a 1.96-point decrease in childhood IQ for each log-unit increase in BPF concentration, whereas no association was found with BPA nor BPS.^56^ Another recent study of 1077 mother-neonate pairs in China linked high prenatal BPF levels to an increased risk for developmental delays in gross and fine motor function, and problem solving.^57^

Epidemiological studies have found links between early life phthalate exposure and neurodevelopmental outcomes with inconsistent results. Recently, Vilmand et al., published findings from the Odence Child Cohort in Denmark, reporting an inverse relationship between both prenatal and postnatal exposure to phthalates and IQ in 7-year-old children (*n* = 585).^58^ Third trimester exposure to certain phthalates was associated with higher externalizing problem scores in 122 school-age children in Taiwan.^59^ However, the Center for the Health Assessment of Mothers and Children of Salinas (CHAMACOS) study, a cohort of low-income Mexican Americans, revealed mostly null associations between prenatal phthalate exposure and a broad range of measures of executive and cognitive function, with the exception of a weak association with internalizing and externalizing problems in adolescents.^60^ A systemic review without meta-analysis published in 2015 concluded that prenatal phthalate exposure had an adverse effect on cognitive function and behavior in children^61^; whereas a more recent systemic review and meta-analysis found that evidence for a relationship between early life phthalate exposure and neurodevelopmental outcomes was lacking or weak.^62^

In rural Costa Rica evidence from 355 mother-child dyads living in close proximity to banana plantations, prenatal exposure to pesticides and fungicides used in agriculture and government vector control programs was related to poor performance on cognitive, language and motor skills tests at one year of age.^63^ In a study of 265 children in New York City, FSIQ and working memory at 7 years of age declined with high cord blood levels of a chlorinated organophosphate insecticide that was banned in the USA in 2021.^64^ Data from a prospective birth cohort in urban China (*n* = 2782) demonstrated an inverse association between neurodevelopmental and psychomotor scores at 2 years of age and *in utero* exposure to organophosphate insecticides, pryethroids, and neonicotinoid insecticides with the strongest association with first trimester exposure in boys.^65^ Systemic reviews and meta-analyses have concluded consistent evidence for a relationship between *in utero* exposure to organophosphate pesticides and childhood mental health, ADHD, and autism, whereas evidence for an impact of postnatal exposure is less consistent.^66–68^

## Childhood metabolic health

Many EDCs are thought to be obesogens and have been investigated for their impact on childhood obesity and risk for cardiometabolic disease. Data from the CHAMACOS cohort demonstrated an association between prenatal exposure to monoethyl phthalate (MEP) and monocarboxy-isononly phthalate (MCNP) and BMI at 5 years of age.^69^ No association was found between prenatal exposure to BPA, parabens, or the other phthalates measured including DEHP.^69,70^ However, a prospective cohort study with 402 dyads found a relationship between prenatal BPA exposure and childhood obesity at 14 months and 4 years of age.^71^ Another prospective cohort study found high concentration of phthalate metabolites in umbilical blood to be predictive of BMI and head circumferences measured over the first 11 months of life, but with a limited sample size.^72^ Overall, current evidence for a link between childhood obesity and prenatal exposure to bisphenols and phthalates is lacking, while there is stronger evidence supporting a relationship between childhood weight and postnatal exposure. Childhood exposure to phthalates and bisphenols is positively associated with measures of obesity including BMI and waist circumference and hip circumference with sex potentially acting as a modifier.^73^

PFAS have been investigated for their association with metabolic disease. A systematic review and meta-analysis quantifying obesity measures at birth found that prenatal PFAS exposure was not associated with BMI or waist circumference.^74^ However, in 1391 mother-child pairs from the ECHO cohort, PFAS exposure during pregnancy was found to slightly increase the risk of overweight/obesity in the offspring between 2 to 5 years of age.^75^ Overall, there is limited evidence supporting the association between prenatal PFAS exposure and childhood metabolic disease.

A systematic review and meta-analysis found a significant association between childhood BMI and prenatal exposure to the organochlorine pesticides dichlorodiphenyldichloroethylene (DDE) and hexachlorobenzene (HCB), while a separate systematic review concluded that prenatal exposure to organochlorine pesticides was associated with insulin resistance and overweight.^76^ A longitudinal study found higher serum organochloride levels were associated with increased BMI at 12 and 24 months of age.^42^ A prospective study of 247 children born to mothers with higher occupational exposure to pesticides were found to have lower birthweight followed by increased BMI later in life at six to eleven years of age when compared to unexposed children.^77^ Overall, the evidence supports that prenatal pesticide exposure is associated with BMI and risk of overweight in childhood.

## Conclusion

**Fig. 2:**
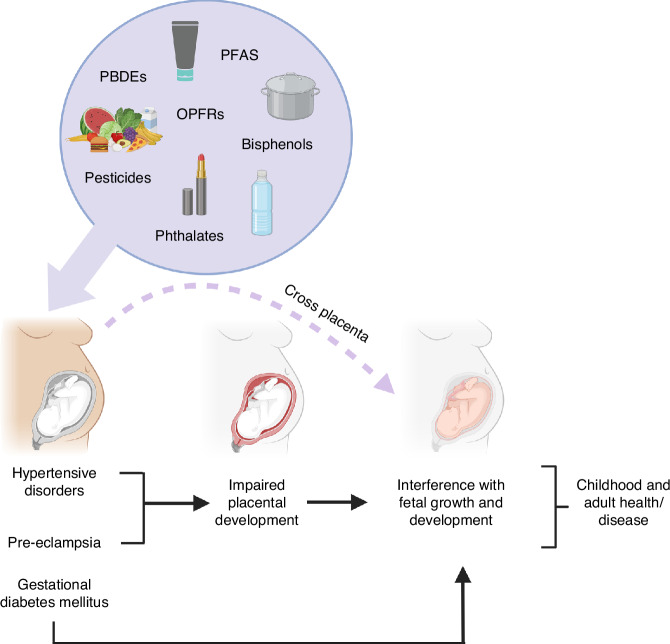
Mechanisms linking maternal exposure to chemical pollutants to childhood and adult disease in the offspring. Maternal exposure to chemical pollutants during pregnancy may increase the risk for antenatal disorders or compromise placental development, leading to altered growth and development of the fetus. Many chemical pollutants have been shown to cross the placenta and can thereby interfere with critical windows of organ development. Image made in BioRender.

The DOHaD field has shed light on the importance of early life development in shaping lifelong health and disease risk. Today, one of the most significant threats to the developmental potential of children worldwide is the increasing contamination of our environment by anthropogenic chemicals. Maternal exposure during pregnancy may interfere with fetal development through compromising maternal health during pregnancy, impairing placental adaptations or crossing the placenta (Fig. 2). While there exists strong evidence demonstrating adverse pregnancy outcomes such as hypertensive disorders of pregnancy, gestational diabetes, and low infant birth weight to be associated with exposure to persistent organic chemicals and other historical chemicals now heavily regulated, results of studies investigating phthalates and bisphenols are inconclusive. Evidence showing neurodevelopmental toxicity of flame retardants and organophosphate pesticides is strong, particularly when exposure occurs prenatally. Prenatal exposure to bisphenols is most consistently associated with behavioral problems in children, while evidence linking neurodevelopmental outcomes to prenatal or postnatal phthalate exposure remains weak. Childhood metabolic health is more often correlated with postnatal exposure to bisphenols and phthalates, whereas prenatal exposure has yielded inconsistent results. While animal studies are useful for establishing causality and identifying mechanisms, human studies investigating health impacts of environmental pollutants rely on observational studies, which are prone to residual confounding bias. The risk for misclassification of exposure levels based on single time-point urine or blood samples is higher for ubiquitous, but rapidly metabolized pollutants like phthalates and bisphenols.
